# Editorial: Canine Intervertebral Disc Disease: The Current State of Knowledge

**DOI:** 10.3389/fvets.2021.656764

**Published:** 2021-03-16

**Authors:** Natasha J. Olby, Andrea Tipold

**Affiliations:** ^1^Department of Clinical Sciences, North Carolina State University, Raleigh, NC, United States; ^2^Department of Small Animal Medicine and Surgery, University of Veterinary Medicine Hannover, Hannover, Germany

**Keywords:** intervertebral disc disease, canine, spinal cord injury, disc extrusion, disc degeneration

Intervertebral disc disease in dogs was first described in the late 1800s with a case report in which the author puzzled over whether the cartilaginous mass within the vertebral canal of a dachshund could be related to the underlying intervertebral disc. Degeneration of intervertebral discs was recognized and described in detail in the 1940s and 1950s and since that time the seminal work of Hansen has imprinted types I and II intervertebral disc disease on every veterinarian. Due to the popularity of certain breeds of dog, intervertebral disc disease is by far the most common cause of canine paralysis and is treated on a daily basis by veterinarians. More recently, the advent of accessible magnetic resonance imaging and dramatic advances in molecular tools have led to expansion and refining of our categorization of intervertebral disc disease, understanding of the genetic risk factors, and improved ability to describe, quantify, and treat the associated spinal cord injury.

This collection of 10 articles takes a holistic look at what we now know about intervertebral disc disease. One of the most exciting advances in the field is the identification of a strong genetic risk factor in chondrodystrophic breeds of dog. The finding of an expressed FGF4 retrogene on chromosome 12 in these breeds of dog is described in detail by Dickinson and Bannasch. This paper documents the discovery of the variant in Nova Scotia Duck Tolling Retrievers and then its validation across other breeds. Mechanisms by which overexpression of FGF4 might exert its influence on the developing intervertebral disc are presented and discussed.

The impact of this finding has been incorporated into a discussion of the classification of intervertebral disc disease in a paper by Fenn et al. This paper also reviews the numerous types of acute intervertebral disc extrusion that are now well-recognized thanks to MRI and incorporates them into a classification scheme. This scheme classifies disc herniation according to the degenerative process underlying it, and takes into account genetic, imaging, and pathological findings. Diagnosis of intervertebral disc degeneration and herniation using imaging is presented in careful detail in a review paper by da Costa et al.. This paper describes key findings reported in the literature using plain radiography, myelography, and computed tomography but then updates the reader with a description of the most current magnetic resonance imaging (MRI) research. Specific imaging features of different types of IVDD are described and illustrated in detail and advanced MR imaging techniques are explained and their potential for future clinical utility is discussed.

Establishing prognosis for recovery, and understanding factors that alter prognosis is important for owners and veterinarians, and can play a role in optimizing case allocation in clinical trials. There are abundant data on prognosis for intervertebral disc extrusions and this literature is summarized and presented in tabular form in a paper on prognosis by Olby et al. Signalment, clinical presentation, imaging, plasma, and CSF biomarkers are all discussed. These data are taken into consideration in a paper describing Current Approaches to the Management of Acute Thoracolumbar Disc Extrusion by Moore et al. This paper is careful to identify knowledge gaps where high level evidence is lacking and draws on published evidence available to make recommendations on surgical vs. conservative management, timing, type and extent of surgery, the role of fenestration, and adjunctive neuroprotective and post-operative care.

Given the high frequency of intervertebral disc-induced spinal cord injury, there is much interest in performing well-designed clinical trials to identify the best therapy for this condition. The many different considerations required to design a clinical trial are described by Jeffery et al.. This article explains the pros and cons of different styles of trial and explains how the information generated might be used clinically. Readers of the article by Lewis et al. on emerging and adjunctive therapies for disc-induced spinal cord injury will learn of the myriad of different therapies that have been investigated for this condition. The evidence for efficacy of locally applied therapies such as laser and pulsed electromagnetic therapy, different pharmaceuticals, cellular therapies, adjunctive surgical techniques and rehabilitation among others are all presented with a careful consideration of the evidence available.

The consequences of acute disc extrusion on the spinal cord are described by Spitzbarth et al. in a well-illustrated paper on the pathology of disc induced spinal cord injury. This paper gathers together detailed descriptions of histopathological changes and highlights what is known on the molecular processes underlying these changes with a particular emphasis on the complex inflammatory response. In addition, the unique changes that occur with progressive myelomalacia are described. Less frequently discussed, but extremely important outcomes of spinal cord injury are addressed in two articles, by Lewis et al. and Granger et al. The article by Lewis et al. takes a careful look at the recovery of walking in dogs that do not recover pain perception following severe, disc induced spinal cord injuries. This paper explains what is known about the circuitry behind locomotion and discusses the evidence presented for the mechanism of recovery of walking in this population of dogs. The article by Granger et al. focuses on urinary and fecal incontinence, a consequence of spinal cord injury that is particularly difficult to manage. The pathways involved in normal control are explained and the impact of spinal cord injury on those pathways is discussed in detail. The frequency and circumstances in which there are long term issues with incontinence are described and methods of management are discussed.

The purpose of this topic is to provide a detailed summary of what is known about intervertebral disc disease in dogs, harnessing the great strides made in recent years. The majority of these articles were written by members of the Canine Spinal Cord Injury Consortium, CANSORT SCI, a group of veterinarians actively involved in the field of spinal cord injury research. These papers collectively have provided a clear summary of the genetic, imaging, and histopathological characteristics of intervertebral disc disease, allowing the development of a new classification system. The detailed summaries of the body of evidence for a wide range of different therapies, and of the factors that influence recovery of motor and autonomic function enable the reader to weigh the most appropriate therapeutic approach for their cases and to identify unexpected outcomes that might need intervention. Our hope is that this collection will provide an accessible and efficient means of updating knowledge of this extremely broad topic and prove to be a springboard from which new ideas can flow.

## Author Contributions

NO and AT have overseen the entire Research Topic. NO wrote the original draft of this editorial with editing input and advice from AT. All authors contributed to the article and approved the submitted version.

## Conflict of Interest

The authors declare that the research was conducted in the absence of any commercial or financial relationships that could be construed as a potential conflict of interest.

